# Dental implants in patients with epidermolysis bullosa: a systematic review

**DOI:** 10.1007/s10006-019-00802-0

**Published:** 2019-10-28

**Authors:** Bruno Ramos Chrcanovic, Ricardo Santiago Gomez

**Affiliations:** 1 Department of Prosthodontics, Faculty of Odontology, Malmö University, Carl Gustafs väg 34, SE-214 21 Malmö, Sweden; 2 Department of Oral Surgery and Pathology, School of Dentistry, Universidade Federal de Minas Gerais, Belo Horizonte, Brazil

**Keywords:** Epidermolysis bullosa, Dental implants, Survival, Complications, Recommendations

## Abstract

**Purpose:**

To integrate the available data published on patients with epidermolysis bullosa (EB) rehabilitated with dental implants, as well as to review the recommendations for EB patients receiving implants.

**Methods:**

An electronic search was undertaken in February 2019 in five databases.

**Results:**

Sixteen publications were included, reporting 28 patients with EB, rehabilitated with 161 dental implants. The mean ± SD patients’ age at implant surgery was 34.7 ± 12.1 years (range, 13–56). Only one implant was placed in the molar region, all other implants were placed in the incisor, canine, and premolar regions. Patients received a mean ± SD of 5.8 ± 2.8 implants (range, 2–11). Most of the patients received implant-supported fixed prostheses (fixed partial 14.3%, fixed full-arch 60.7%, overdenture 25%). Implant and prosthesis failure rates were 1.3% and 0%, respectively. The two implant failures were detected before or at the abutment connection. The mean ± SD follow-up time was of 39.2 ± 24.5 months (range, 6–111). The EB patient quality-of-life improved considerably as a result of treatment with dental implants. There is a series of dental care considerations that should be followed to smooth the implant treatment in EB patients.

**Conclusions:**

The dental implant failure rate in EB patients seems to be very low, although the few cases reported in the literature were followed up for a short mean period, i.e., just a little bit longer than 3 years. More cases followed up for a long period are needed in order to be able to make a more reliable prognosis for the long-term oral rehabilitation of EB patients with dental implants.

## Introduction

Epidermolysis bullosa (EB) is a rare, inherited, recessive disease of the skin and mucosal membranes, characterized by trauma-induced bullae, pseudosyndactyly of hands and feet, and scar formation [1]. The most common oral manifestation of EB is blistering, but it can also include the appearance of milia (milia are tiny—1 to 4 mm—benign white papules that usually are located on the palate and correspond histologically to keratin-filled cysts), microstomia, ankyloglossia (adherence of the tongue to the floor of the mouth), severe periodontal disease, enamel hypoplasia, dental caries, and atrophy of the maxilla with mandibular prognathism [2–4]. The wearing of removable dentures for patients with EB is very difficult due to the fragility of the oral mucosa, as pressure and micromovements by removable dentures cause irritation, ulcerations, and pain [1]. Implant-supported restorations may minimize trauma to the oral mucosa, improving the quality of life of patients with EB. In order to verify whether EB has any effect on the treatment with dental implants, the aim of the present study was to integrate the available data published in the literature on patients with EB rehabilitated with dental implants.

## Materials and methods

This study followed the PRISMA Statement guidelines [5].

### Search strategies

An electronic search without time restrictions was undertaken in February 2019 in the following databases: PubMed/Medline, Web of Science, Science Direct, J-Stage, and Lilacs. The following terms were used in the search strategies:

#### Epidermolysis bullosa AND (dental implant OR oral implant)

Google Scholar was also checked. A manual search of all related oral pathology, maxillofacial, and specialist dental and oral journals was performed. The reference list of the identified studies and the relevant reviews on the subject were also checked for possible additional studies.

### Inclusion and criteria

Eligibility criteria included publications reporting cases of patients with EB and rehabilitated with dental implants.

### Study selection

The titles and abstracts of all reports identified through the electronic searches were read independently by the authors. For studies appearing to meet the inclusion criteria, or for which there were insufficient data in the title and abstract to make a clear decision, the full report was obtained. Disagreements were resolved by discussion between the authors.

### Data extraction

The review authors independently extracted data using specially designed data extraction forms. Any disagreements were resolved by discussion. For each of the identified studies included, the following data were then extracted in a standard form, when available: patient’s sex and age, implant location (maxilla/mandible, region of incisors, canines, premolars, molars), grafting procedures, implant healing time, type of prosthetic rehabilitation, implant failure, time to failure, and follow-up period. Contact with authors for possible missing data was performed.

### Analyses

A descriptive analysis was performed based on mean, standard deviation (SD), and percentage values. The level of statistical significance was set at *P* < 0.05. All data were analyzed using IBM SPSS Statistics for Windows, version 25.0 (IBM Corp., Armonk, NY, USA).

## Results

### Literature search

**Fig. 1:**
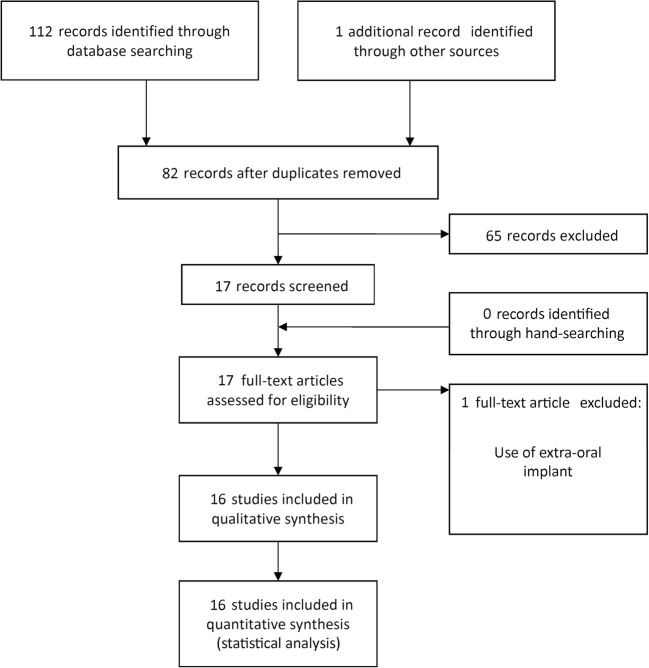
Study screening process

The study selection process is summarized in Fig. 1. The search strategy in the databases resulted in 112 papers; 1 additional eligible paper was found in Google Scholar, and no papers through hand-searching. At the end, a total of 16 publications were included [6–21].

### Description of the studies and analyses

Table 1 presents demographic and clinical features of patients with EB rehabilitated with dental implants described in the literature. There were 28 patients (8 males, 19 females, 1 unknown) and their mean ± SD age at the time of implant placement was 34.7 ± 12.1 years (range, 13–56). All patients were reported to have microstomia and had antecedents of bleeding blisters, rampant dental caries, and loss of dentition. Many patients were reported with ankyloglossia and obliteration of the oral vestibule. Only one patient had Kindler syndrome, one of the major group types of EB [22]. These patients received 161 dental implants, and about half of the implants were placed in each jaw. The patients received a mean ± SD of 5.8 ± 2.8 implants (range, 2–11). About 83% of the implants were placed under local anesthesia. About 15% of the implants were placed with bone augmentation procedures or in grafted sites. Only one implant was placed in the molar region of the jaws, for the support of a partial fixed prosthesis, in a patient that still had 18 natural teeth. All other implants were placed in the incisor, canine, and premolar regions.

**Table 1:** Demographic and clinical features of patients with epidermolysis bullosa rehabilitated with dental implants described in the literature

Variables	
Patients (*n*)	28
Male (%)/female (%)^a^	8 (29.6)/19 (70.4)
Age (years), mean ± SD (min–max)	34.7 ± 12.1 (13–56)
Implants (*n*)	161
Male (%)/female (%)^a^	57 (36.5)/99 (63.5)
Implants per patient, mean ± SD (min–max)	5.8 ± 2.8 (2–11)
Maxilla (%)/mandible (%)	83 (51.6)/78 (48.4)
Failure/total (%)^b^	2/156 (1.3)^c^
Healing time maxilla, mean ± SD (min–max)	5.7 ± 0.6 (4–6)
Healing time mandible, mean ± SD (min–max)	3.6 ± 1.3 (0–6)
Use of osteotome for placement in maxilla (%)	56/72 (77.8)^d^
Placement under local (%)/general anesthesia (%)	125 (82.8)/26 (17.2)^e^
Augmentation procedures/total (%)	24/161 (14.9)
Position (%)	
Incisor	60 (39.7)
Canine	41 (27.2)
Premolar	49 (32.4)
Molar	1 (0.7)
Not available	10
Prosthetic rehabilitation (%)	
Fixed partial	4 (14.3)
Full-arch	17 (60.7)
Overdenture	7 (25.0)
Failure	0/28 (0)
Follow-up time^f^ (months), mean ± SD (min–max)	39.2 ± 24.5 (6–111; *n* = 151)

*SD*, standard deviation

^a^Sex was not informed for 1 patient (5 implants)

^b^There was no information on survival for 1 patient (5 implants)

^c^Both implant failures occurred before or at the abutment connection

^d^Information not available for 11 maxillary implants

^e^Information not available for 2 patients (10 implants)

^f^There was no precise information on follow-up time for 2 patients (10 implants)

Only 2 (1.3%) out of 156 implants with follow-up information failed. Both failures were detected before or at the abutment connection surgery (primary failures), one (out of five implants) in the maxilla and the other one (out of four implants) in the mandible, but in distinct patients. In both patients, the loss of only one implant did not compromise the initial plan of rehabilitating the patient with a fixed full-arch prosthesis. Most of the patients received fixed prostheses, either partial (14.3%) or full-arch (60.7%), and 1/4 of the patients received overdentures. The fixed full-arch prostheses were all short-expanded; none of them had more than 10 prosthetic teeth. None of these prostheses failed. The patients were followed up for a mean ± SD time of 39.2 ± 24.5 months (range, 6–111).

## Discussion

The use of implant therapy requires consideration of potential benefits to be gained from the therapy. To better appreciate this potential, the present study aimed to integrate the available data published in the literature on patients with EB rehabilitated with dental implants. A review of rare cases and condition provides information that allows professionals to make improved decisions and refine treatment plans to optimize clinical outcomes [23–27].

In relation to a previous recent review on the subject [1], the present study performed a more careful systematic search of the literature, thus resulting in a greater number of included studies. One flaw of this previous review was the fact that duplicated cases from several publications of the same research group [17, 18, 20, 21] were considered as distinct patients, artificially increasing the number of patients and implants. Moreover, the present study performed a more in-depth and detailed statistical analysis of the compilation of included studies.

In general, it was observed that EB patients rehabilitated with implant-supported prostheses were still presenting blisters and ulcerations, mainly as a result of stiff food particles and vigorous brushing, as well as a result of attrition in zones of contact, in the cases which the patient was rehabilitated with overdentures. Still, most studies reported that the EB patient quality-of-life improved considerably as a result of treatment with dental implants. It was mentioned by some [18, 19] that before the rehabilitation with the implant-supported restorations, the patients were no able to chew properly, and all food had to be swallowed in the *purée* form to avoid esophageal ulceration. The patients were then able to chew and swallow well-ground food bolus after prosthesis rehabilitation. In another report, the patient reported that he could masticate all types of foods and started gaining weight [13].

The implant failure rate was only 1.3%. Both implant failures were detected before or at the abutment connection surgery (primary failures), which agrees with the results of clinical studies showing that a considerable number of implant failures occur early [28, 29], regardless of whether patients are followed up for a long period [30]. However, the mean follow-up was just a little bit longer than 3 years, which is very short. Thus, it is expected that the implant failure rate is underestimated, as a longer follow-up period can lead to an increase in the failure rate. The same observation is valid for the prosthetic failure rate.

It is not surprising that edentulous patients were rehabilitated with short-expand fixed full-arch prostheses, and none of the implants were placed in the molar region of the jaws. The most posterior implants in these cases were placed in the premolar area, as there were difficulties in accessing the posterior regions of the mouth due to microstomia. The only implant placed in the molar region of the jaws was used to support a 3-unit partial fixed prosthesis. Microstomia in EB patients is caused by a constant process of blister formation and healing that result in scarring in the commissures and loss of vestibular space [31]. On rupture, bullae leave painful ulcers, followed by scarring and tissue contraction [4]. Therefore, in order to minimize manipulation of the oral soft tissues, short dental arch rehabilitation with fixed prostheses is the rehabilitation technique advised [13, 15, 17].

More than 3/4 of the implant surgical sites in the maxilla were prepared by using osteotomes. The reason for this is that conventional drilling procedure has the potential to destroy the entire residual alveolar process, thus complicating primary stability of the implants [21]. Others suggested the use of drills of smaller diameter [15].

It is suggested that general anesthesia should not be regularly used in EB patients because of the risk of esophageal bullae during intubation [18]. Only three patients were operated under general anesthesia [8, 13, 15], but these studies failed to report the presence of complications. In case local anesthesia is used, it is recommended to inject it deeply into the tissues at a sufficiently slow rate to prevent tissue distortion, which can cause mechanical tissue separation and blistering [32].

Some other recommendations for the treatment of EB patients with dental implants were mentioned and could be useful, such as the following: (a) aspiration should be done with an aspirator in contact with bone or teeth, not soft tissues, in order to avoid minor trauma to the oral mucosa [18]; (b) lubrication of patient’s lips with, for example, petroleum jelly or glycerin, to minimize minor trauma [32]; (c) whenever possible, incisions with flaps should be minimized, to avoid the production of blisters during surgery [18]. For a more complete and detailed list of recommendations, not only for the treatment with dental implants, the article of Feijoo et al. [2] should be consulted.

The limitations of the present review include the fact that all included studies were retrospective case reports, which inherently result in errors, with incomplete records. The use of precise information on every variable would improve the quality of the statistical analyses [33]. Secondly, many of the published cases had a short follow-up. The report of more cases followed up for many years would help us to make a more reliable prognosis for the oral rehabilitation of EB patients with dental implants in the long term.

## Conclusions

The dental implant failure rate in EB patients seems to be very low, although the few cases reported in the literature were followed up for a short mean period, i.e., just a little bit longer than 3 years. More cases followed up for a long period are needed in order to be able to make a more reliable prognosis for the long-term oral rehabilitation of EB patients with dental implants.
